# Pharmacological Effects of Polyphenol Phytochemicals on the Intestinal Inflammation via Targeting TLR4/NF-κB Signaling Pathway

**DOI:** 10.3390/ijms23136939

**Published:** 2022-06-22

**Authors:** Caiyun Yu, Dong Wang, Zaibin Yang, Tian Wang

**Affiliations:** 1 College of Animal Sciences and Technology, Nanjing Agricultural University, No. 1 Weigang Street, Nanjing 210095, China; yucaiyun1129@163.com; 2 Heilongjiang Key Laboratory of Experimental Animals and Comparative Medicine, College of Veterinary Medicine, Northeast Agricultural University, Harbin 150000, China; wangd931107@163.com; 3 College of Animal Sciences and Technology, Shandong Agricultural University, No. 61 Daizong Street, Tai’an 271018, China; yangzb@sdau.edu.cn

**Keywords:** polyphenols, TLR4/NF-κB signaling pathway, intestinal inflammation

## Abstract

TLR4/NF-κB is a key inflammatory signaling transduction pathway, closely involved in cell differentiation, proliferation, apoptosis, and pro-inflammatory response. Toll like receptor 4 (TLR4), the first mammalian TLR to be characterized, is the innate immune receptor that plays a key role in inflammatory signal transductions. Nuclear factor kappa B (NF-κB), the TLR4 downstream, is the key to accounting for the expression of multiple genes involved in inflammatory responses, such as pro-inflammatory cytokines. Inflammatory bowel disease (IBD) in humans is a chronic inflammatory disease with high incidence and prevalence worldwide. Targeting the TLR4/NF-κB signaling pathway might be an effective strategy to alleviate intestinal inflammation. Polyphenol phytochemicals have shown noticeable alleviative effects by acting on the TLR4/NF-κB signaling pathway in intestinal inflammation. This review summarizes the pharmacological effects of more than 20 kinds of polyphenols on intestinal inflammation via targeting the TLR4/NF-κB signaling pathway. We expected that polyphenol phytochemicals targeting the TLR4/NF-κB signaling pathway might be an effective approach to treat IBD in future clinical research applications.

## 1. Introduction

Inflammatory bowel disease (IBD), including mainly Crohn’s disease and ulcerative colitis (UC), is a chronic intestinal inflammation characterized by bellyache, malabsorption, diarrhea, general malaise, etc. [1]. The incidence areas of CD can occur throughout the gastrointestinal tract, whereas the main incidence area of UC is the colon and rectum [2]. Approximately 3 million adults in the United States were diagnosed with IBD in 2015, and the incidence rate in 2030 is predicted to increase to 4–6 times that [3]. The incidence rate of IBD in China is 3.44%, ranking the highest in Asia [4]. To date, preclinical models of IBD are widely established to explore the pathogenesis and therapy. Furthermore, 2,4,6-trinitrobenzene sulfonic acid (TNBS) and dextran sulfate sodium (DSS) models have been largely employed.

Inflammatory signaling pathways play a crucial role in the treatment of inflammatory disease. Several external stimuli can activate toll-like receptor 4 (TLR4) and downstream nuclear factor kappa B (NF-κB) pathway, also promoting the production of inflammatory cytokines, subsequently provoking the inflammatory response [5]. As shown, there is strong evidence of the upregulation of TLR4/NF-κB and MAPK signaling in IBD [6,7]. IBD patients are commonly treated with medicine therapy but this gives rise to a lot of side effects. Therefore, there are well recognized requirements for new and safe strategies for IBD treatment. On that basis, accumulating studies demonstrated the pharmaceutical effects of polyphenols on the IBD. Polyphenols are secondary metabolites of plants that normally contain at least one or more hydroxyl group-linked benzene rings [8]. In the past, accumulating evidences suggested that polyphenols are potential sources of alternative medications to treat the oxidative stress and inflammation associated with degenerative diseases, such as diabetes mellitus (DM), rheumatoid arthritis (RA), and cardiovascular disease [9]. More importantly, a recent study showed that polyphenol extract of *Moringa oleifera* containing astragalin, chlorogenic acid, isoquercitrin, kaempferitrin, luteolin, quercetin, and rutin could alleviate colonic inflammation in DSS-treated mice associated with the NF-κB signaling pathway [10], indicating the anti-inflammatory potential of polyphenols on intestinal diseases. The small intestine plays a key role in the digestion and absorption of nutrients, including carbohydrates, proteins, and lipids. To date, the gastrointestinal tract has been considered as a potential research hotspot that is associated with inflammation induced by pathogens, toxins, and external stimulus [11]. Increased attention has been paid to the link between the polyphenols and intestinal inflammation. Increased intestinal inflammation is largely driven by activation of the TLR4/NF-κB signaling pathway [6]. It is worth noting that numerous studies have been conducted to date on the anti-inflammatory effects of polyphenols, in both in vitro and in vivo multiple inflammatory models, but few studies have addressed the specific effect and mechanisms of polyphenols on intestinal inflammation. However, although various models of severe intestinal inflammation were used, these pathologies share common inflammatory processes and mechanisms. In this regard, the present review will focus on recent advances in the intestinal anti-inflammatory properties of polyphenols which link the TLR4/NF-κB-mediated signaling pathways in both in vitro and in vivo intestinal inflammatory models. Polyphenols could contribute, as adjuvant, or preventive approaches, to the treatment of chronic inflammatory diseases.

## 2. TLR4 Signaling Pathways

The innate immune system constitutes the first line of host defense against extraneous pathogen invasion, including bacteria, viruses, yeasts, and fungi. Transmembrane receptors designated toll-like receptors (TLRs) belonging to members of pattern recognition receptors (PRRs) play a key role in recognizing invading microbial pathogens and inducing innate immune responses for the host defense [12]. They are expressed on multiple immune cells, including B cells, dendritic cells, macrophages, specific types of T cells, and even on non-immune cells such as intestinal epithelial cells [13]. TLRs are type I transmembrane glycoproteins constituted by an extracellular N-terminal domain of leucine-rich repeats and an intracellular C-terminal domain similar to that of the interleukin 1 receptor (IL-1R), thus designated as toll/interleukin 1 receptor (TIR) domain, which is responsible for downstream signal transduction [13,14].

TLR4, one class of TLRs, is thought to play a crucial role in intestinal inflammatory diseases [6]. It can lead to the maturation of dendritic cells and differentiation of helper T cell (Th) 1 and Th2 [7]. Moreover, it can induce the differentiation of macrophages to an M1 phenotype, thereby producing pro-inflammatory cytokines [15]. Upon activation, TLR4 dimerizes and triggers two major signaling cascades, myeloid differential factor 88 (MyD88)-dependent and toll/interleukin 1 receptor domain-containing adaptor inducing interferon-beta (TRIF)-dependent pathways, which result in the downstream activation of NF-κB and mitogen-activated protein kinases (MAPKs) and induction of various pro-inflammatory gene products, including cytokines and inflammation related enzymes [14,16].

The MyD88-dependent pathway begins with the cytoplasmic TIR domain [17]. Upon MyD88 activation associated with TIR domain-containing adaptor protein (TIRAP), the autophosphorylation of IL-1 receptor-associated kinase (IRAK), namely, IRAK1, and IRAK4 was subsequently triggered, and it further temporarily interacts with tumor necrosis factor receptor-associated factor 6 (TRAF6). This activation of IRAK and TRAF6 eventually results in the phosphorylation and degradation of NF-kappa-B inhibitor alpha (IκBα), and the following translocation of NF-κB into the nucleus [14,18]. In addition, TRAF6 can stimulate MAPKs, namely, p38, extracellular signal regulated kinase (ERK), c-Jun N-terminal kinase (JNK), and the subsequent activation of the activator protein-1 (AP-1) [19]. Next, the activation of NF-κB and MAPK can induce inflammatory responses through the activation of inflammation related enzymes, such as inducible nitric oxidase synthase (iNOS), cyclooxygenase 2 (COX-2), and pro-inflammatory cytokines secretion, such as interleukin-1β (IL-1β), IL-6, IL-8, tumor necrosis factor-α (TNF-α), and others [19]. On the other hand, the TRIF-dependent pathway is also confirmed to trigger after TLR4 activation. It primarily recruits TRIF and leads to the ubiquitination of TRAF6, which induces TANK-binding kinase 1 (TBK1) combining to I-kappa-B kinase epsilon (IKKε, the inhibitor of NF-κB). Later, the transcription factor interferon regulatory factor 3 (IRF3) is phosphorylated and activated by the TBK1-IKKϵ complex, finally driving the transcription of interferon-alpha (IFN-α) and IFN-β [20,21].

## 3. TLR4 and NF-κB in the Development of Inflammatory Bowel Disease

As mentioned earlier, IBD is a chronic, relapsing, and lifelong disease that has been a worldwide threat to healthcare with increasing incidence and prevalence. More importantly, there is strong evidence that TLRs, and TLR-activated signaling pathways, are involved in the pathogenesis of IBD [7,22]. TLRs not only play a crucial role in innate immunity, but also critically modulate adaptive immunity, such as T cell activation. There is disequilibrium between T regulatory cells (Tregs) and effector T cells in patients with IBD. This implies that when Tregs’ function of inhibiting effector T cells, such as Th1, Th2, Th17, and NKT cells, is suppressed due to TLR-induced over immune responses, IBD will become out of control [23,24]. In addition, TLRs act as the bridge between immune response to microbes in the gut, thus giving rise to IBD [7]. That is, the innate inflammatory response can result in dysbiosis of the intestinal microbiota, leading to host metabolic dysfunction. In this respect, TLRs can mediate the interactions between the host immunity and intestinal microbiota. Taken together, TLRs are a potential molecular mechanism in the development of IBD due to controlling the immune response and disordering the intestinal microbiome.

Among all TLRs, the TLR4 is the first verified TLR in the mammalian system and the receptor of lipopolysaccharide (LPS) in Gram-negative bacteria. Under normal physiological conditions, TLR4 is expressed at a low level in intestinal epithelial cells [25]. However, the TLR4 is expressed at high levels in the intestinal epithelium of patients with active UC, indicating that TLR4 might be involved in the development of UC. NF-κB is the final transcription factor of the TLR4 signaling pathway. The NF-κB signaling pathway plays a pivotal role in promoting the development of intestinal diseases via regulation of transcription and translation of inflammatory mediators, such as pro-inflammatory cytokines [26]. NF-κB is formed by five important proteins, including p65 (RelA), p50, p52, c-Rel, and RelB, which exist in cytoplasm as inactive heterodimeric complexes by binding to its inhibitory protein, I kappa B (IκB). P65 is the most representative protein for the regulation and function of NF-κB. Upon activation by various inflammatory stimuli, such as LPS, the activation of the IκB kinase (IkkB) triggers the phosphorylation and degradation of IκBα. Afterwards, nuclear translocation of NF-κB occurs after NF-κB phosphorylation. Upon entering the nucleus, NF-κB binds to DNA and activates the expression of pro-inflammatory genes including cytokines (IL and TNF-α), adhesion molecules, and inducible enzymes (iNOS and COX-2) [27]. Previous study has demonstrated that inflammatory cytokines can induce disturbances in intestinal barrier function, thereby causing intestinal mucosal barrier damage and inflammatory response [22,28]. On the other hand, the high expression of iNOS can lead to high NO production, which participates in the pathology of chronic IBD [29,30]. Cyclo-oxygenases are enzymes that influence many biological processes, ranging from homeostasis to inflammation [31]. There are two cyclo-oxygenases isoforms: the constitutive COX-1 isoform and the inducible COX-2 isoform. Among them, COX-2 induction can be reflected by increased prostaglandin E2 (PGE_2_) levels at the site of inflammation [31,32]. Taken together, regulation of the TLR4/NF-κB-mediated signaling pathway could be novel potential therapeutic strategies against IBD.

## 4. Polyphenols Alleviate Intestinal Inflammation via Modulating the TLR4/NF-κB Signaling Pathway

**Figure 1:**
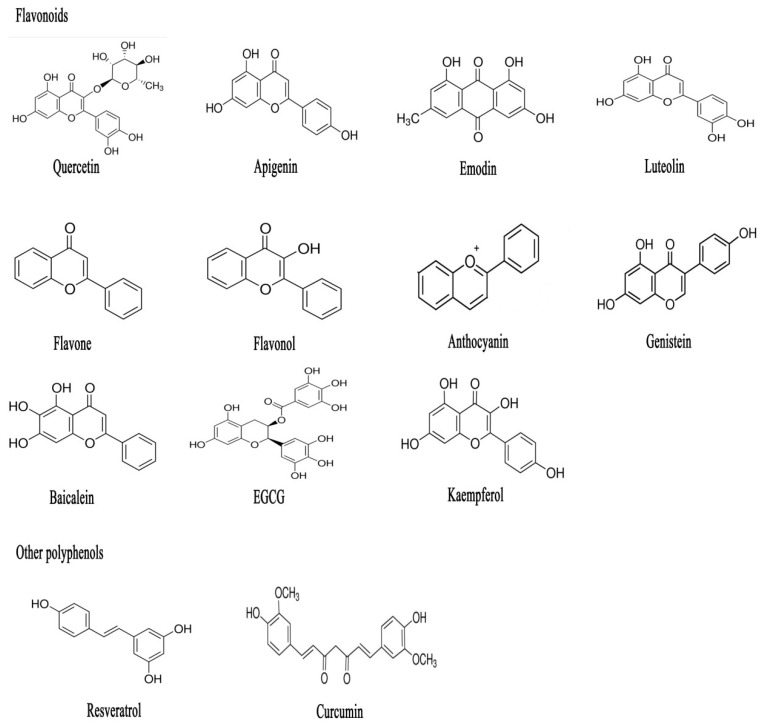
Chemical structure of some of the flavonoids and other polyphenolic compounds featured in this review. Polyphenols can be classified into flavonoids and tannins, alkaloids, terpenoids, and phenylpropanoid. Substantial variation is intuitively observed by distinct chemical substitutions, especially hydroxylation and glycosylation.

**Figure 2:**
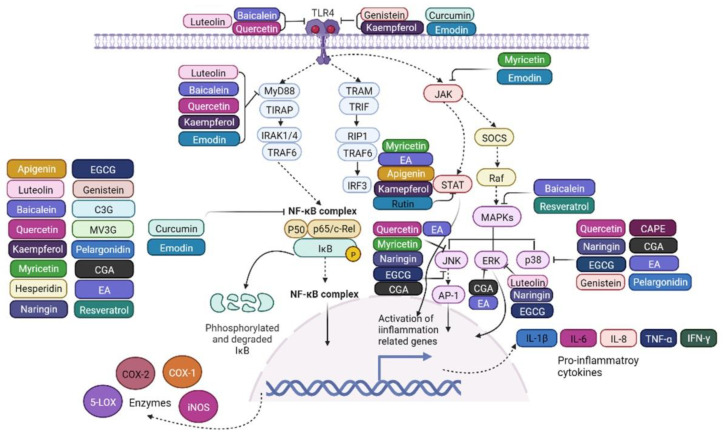
The intestinal anti-inflammatory mediated effects of polyphenols along the TLR4 signaling pathway. EGCG, epigallocatechin-3-gallate; C3G, cyanidin-3-glucoside; MV3G, malvidin 3-glucoside; P3G, pelargonidin-3-O-glucoside; CAPE, caffeic acid phenethyl ester; CGA, chlorogenic acid; EA, ellagic acid. 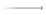 Inhibition; 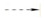 Promotion; 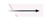 Promotion.

**Figure 3:**
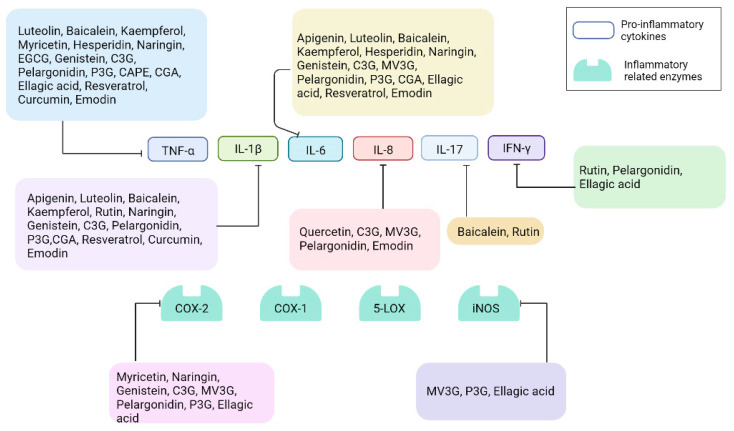
The regulatory inflammatory responses by which polyphenols affect the TLR4-mediated signaling pathway. EGCG, epigallocatechin-3-gallate; C3G, cyanidin-3-glucoside; MV3G, malvidin 3-glucoside; P3G, pelargonidin-3-O-glucoside; CAPE, caffeic acid phenethyl ester; CGA, chlorogenic acid. 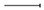 Inhibition.

Polyphenols, widely known as secondary metabolites, are plant-synthesized compounds possessing various biological activities [33,34]. Polyphenols can be classified into flavonoids and tannins, alkaloids, terpenoids, and phenylpropanoid [35]. The chemical structures of some of the polyphenolic compounds are depicted in Figure 1. There are enormous structural variations among these compounds. However, the anti-inflammatory effects of these compounds are consistent in both in vitro and in vivo inflammatory disease models. They become involved in multiple biological processes inside the body, such as radical scavenging and anti-inflammatory processes, as well as cell signaling [9,36,37]. Currently, numerous studies have indicated that phytochemicals may be promising candidates for the treatment of several inflammatory diseases. However, there is a gap in the knowledge of in vitro and in vivo effects although the pharmacokinetics of polyphenols have improved a lot in the last decade [38]. Interestingly, the predominant anti-inflammatory mechanism is attributed to an inhibition of TLR4/NF-κB-mediated signaling pathways and the downregulation of expression of pro-inflammatory mediators [38,39]. Another point worth noting is the evidence that many polyphenols, especially flavonoids, have been studied for their intestinal anti-inflammatory activity associated with inhibition of inflammatory signaling pathways, pro-inflammatory genes expression, and promotion of anti-inflammatory genes expression. In this section, we will discuss, in detail, how polyphenols exert their intestinal anti-inflammatory properties linked with TLR4/NF-κB-mediated signaling pathways in both in vitro and in vivo intestinal inflammatory models (Table 1, Figure 2 and Figure 3).

### 4.1. Flavonoids

Flavonoids are bioactive substances belonging to a family of polyphenolic compounds which exist in natural plants, vegetables, and fruits and consumed in significant amounts as part of the human diet [38]. Flavonoids are recognized as compounds consisting of 3-ring core connected with phenolic hydroxyl groups through three central carbon atoms (Figure 1). According to the connection position of the B-ring (2- or 3-position) and the level of oxidation of the C-ring, flavonoids can be divided into the following six categories: flavonols, flavones, flavanones, anthocyanidins, flavanols, and isoflavones [93]. In addition, there are some flavonoids with unique molecular structure, such as dihydroflavonol and biflavones. The flavone, flavanol, flavanonol, and flavanone families were identified depending on the presence of a 3-OH group and a double bond at 2-position. Compounds with a B ring in the 3-position instead of 2 are isoflavones, of which genistein is the most known substance. Anthocyanidins have a fully aromatized C ring while chalcones are related aryl kenotic compounds with a C opening ring [38,94]. Flavonoids are found in natural plants mainly in glycosylated form. As exhibited in Figure 1, there are substantial structural variations in these compounds, which must affect their biological profile. However, numerous studies provided evidence that there is consistency in the anti-inflammatory effects of these compounds in spite of the structure variations [38]. It has become a research hotspot due to their widely reported bioactive functions and low toxicity, thus they have also become potential therapeutic drugs. González et al. [38] summarized recent advances in the favorable effects of these flavonoids on the treatment of inflammatory diseases, such as rheumatoid arthritis, inflammatory bowel disease, asthma, atherosclerosis, ischaemia-reperfusion, and so on, indicating their outstanding pharmaceutical value with multiple bioactivities. It should be noted that flavonoids have exhibited favorable effects on intestinal tight junction proteins [95,96]. In this regard, accumulating studies have indicated that flavonoids can alleviate intestinal inflammation through inhibiting the activation of the TLR4/NF-κB signaling pathway.

#### 4.1.1. Flavones

##### Apigenin

Apigenin (4′,5,7-trihydroxyflavone) is found in many fruits, herbs, and vegetables, such as celery, parsley, thyme, basil, coriander, and licorice [97]. This flavone has attracted more and more attention due to its anti-inflammatory activities [98,99,100]. Apigenin pre-treatment can ameliorate intestinal damages and restore intestinal barrier integrity in radiation-induced Swiss albino mice, and prevent activation of NF-κB and NF-κB-mediated apoptotic signaling [40]. Apigenin downregulated NF-κB and signal transducer and activator of transcription 3 (STAT3) expression in the LPS-induced colonic epithelial cancer cell [41]. Going downstream, apigenin supplementation exerted protective effects in DSS-induced chronic colitis in mice associated with downregulation of colonic COX-2 and iNOS expression, and IL-1β and TNF-α proinflammatory cytokine [101]. In addition, the intestinal anti-inflammatory effects of apigenin in the treatment of colitis were widely reported [97,102,103].

##### Luteolin

Luteolin (3′,4′,5,7-tetrahydroxy flavonoids) is present in vegetables (carrots, celery, bell peppers), fruits (apple), and herbs (honeysuckle, chrysanthemum, perilla), which has favorable effects on intestinal barrier function. More specifically, luteolin can attenuate ulcerative colitis, suppress rectal cancer, and prevent irinotecan-induced mucositis [104,105,106,107]. From another perspective, luteolin had a notably alleviative effect on intestinal barrier damage induced by decabromodiphenyl ether (BDE-209) in a Caco-2 cell monolayer model through suppressing the phosphorylation of IκBα and the accumulation of NF-κB p50 and ERK expression [42]. Luteolin also relieved DSS-induced colitis in mice, and the mechanism by which is due to the suppression of high mobility group box chromosomal 1 (HMGB1), TLR4, and NF-κB p65 protein levels in the colon [43]. In a co-culture model consisting of intestinal epithelial Caco-2 and macrophage RAW264.7 cells, stimulated with LPS, the addition of luteolin suppressed NF-κB nuclear translocation, followed by reduction of *TNF-α* and *IL-8* mRNA expression, indicating the positive effects of luteolin on gut inflammation [44].

##### Baicalein

Baicalein (5,6,7-trihydroxyflavonoid) is a flavonoid isolated from *Scutellaria baicalensis* Georgi with a variety of pharmacological effects, such as anti-inflammation, anti-oxidative stress, anti-infection, and so on [108]. Radiation-induced enteritis may be an ideal model of gastrointestinal inflammation. Some research revealed that baicalein has a therapeutic effect on radiation-induced intestinal inflammation by accelerating crypt regeneration, attenuating endothelial damage, rebalancing gut microbiota, and inhibiting apoptosis [108,109]. In addition, baicalein administration remarkably suppressed the phosphorylation of NF-κB p65 and IκBα in the colon of TNBS-colitis mice, which was in accordance with the inhibitory effects on the protein expression of TLR4 and MyD88 [45]. In a UC rat model, baicalein can suppress the NF-κB and MAPK pathways to achieve anti-inflammatory effects [46].

#### 4.1.2. Flavonols

##### Quercetin

A plant flavonol, quercetin (3,3′,4′,5,7-pentahydroxyflvanone), present in tea, onions, apples, and red wine, has approved antioxidant, anti-inflammatory, anti-allergic, and anti-virus properties [110], indicating its potential therapeutic application. It was reported that intestinal epithelial (IEC-6) cells pretreated with 5 μmol/L quercetin could resist intestinal barrier dysfunction injury by indomethacin via reducing the JNK phosphorylation and subsequent activation [47]. Pretreatment of quercetin decreased the expression of IL-8 and suppressed the translocation of the p50 subunit of NF-κB into the nucleus in *Vibrio cholerae* induced intestinal epithelial cells [48]. In acute necrotizing pancreatitis disease induced by sodium taurocholate in rats, quercetin blocked intestinal TLR4/MyD88/p38 MAPK pathway and inhibited endoplasmic reticulum stress, thereby ameliorating intestinal barrier disruption and inflammation [49]. A quercetin oxidation metabolite present in onion peel showed protective effects against indomethacin-induced intestinal epithelial barrier dysfunction accompanied by an inhibitory effect on the NF-κB activation and IL-8 secretion [50]. Interestingly, quercetin exhibited a protective effect on mitochondrial dysfunction in intestinal Caco-2 cells [111]. Furthermore, it attenuated intestinal mucosal damage from ischemia-reperfusion injury by inhibiting COX-2 and myeloperoxidase (MPO) expression [112]. Moreover, quercetin was found to be the main active ingredient in a traditional Chinese medicine widely used for UC treatment [113].

##### Kaempferol

Kaempferol, a natural flavonol component isolated from *Cudrania tricuspidata*, is known to have multiple bioactivities, such as anti-inflammatory, anti-oxidant, anti-apoptotic, and anti-cancer effects [114]. Pharmacologically, increasing evidences suggest that kaempferol is an anti-inflammatory compound with activity inhibiting NF-κB, AP-1, and Janus kinase (JAK)/STAT pathways in vitro [115,116]. Lee et al. [115] and Fan et al. [117] revealed that kaempferol can improve barrier function in rat intestinal epithelial cells. A later study also demonstrated that kaempferol can attenuate diquat-induced intestinal dysfunction in intestinal porcine epithelial cells, indicating a functional role of kaempferol in the intestinal barrier [118]. More specifically, kaempferol may be an effective therapeutic agent for IBD treatment reflected by its inhibitory activity on multiple inflammatory pathways and evidenced by blocking NF-κB, I-κB, and STAT phosphorylation, and reducing TLR4 expression, as well as IL-1β, IL-6 and TNF-α secretion induced by LPS in rat intestinal microvascular endothelial cells [51]. Afterwards, the author further demonstrated that kaempferol protected mice from high-fat diet-induced obesity and intestinal inflammation by reducing the activation of the TLR4/NF-κB pathway [52].

##### Rutin

Rutin, quercetin-3-rhamnosyl glucoside, possess a variety of pharmacological effects, such as antioxidant, anti-inflammatory, antibacterial, and radioresistant effects [119]. More importantly, rutin has long been elucidated as the intestinal anti-inflammatory property in acetic acid [120], TNBS [121], and DSS induced rat colitis [122]. Profoundly, rutin inhibited the STAT4-IFN-γ pathway in splenic CD4^+^ cells of mice with CD4^+^CD62L^+^T cells transfer colitis [1]. Afterwards, the author conducted a profound trial to explore whether rutin and its closely related flavonol quercetin can protect against TNBS-induced ileitis and colitis. The results found that intragastric rutin could protect mice against TNBS-induced ileitis, as evidenced by amelioration of anorexia, damage score, body weight loss, and reduction of *IL-1β* and *IL-17* mRNA levels. Colitis induced by TNBS was also ameliorated by rutin which was evidenced by reducing colon thickening, damage score, and the expression of IL-17 and IFN-γ [53].

##### Myricetin and Myricetin-3-O-b-D-Lactose Sodium Salt

Myricetin can be found in many edible plants, such as medicinal herbs, teas, and many fruits, possessing antioxidative, anticarcinogenic, and anti-inflammatory properties [123,124,125]. Myricetin has been proven to improve the intestinal barrier-promoting efficiency in rat IEC-6 cells evidenced by enhanced transepithelial electrical resistance and anti-bacterial effect [126]. Based on that, myricetin further exhibited protective effects on the IEC-6 cells against indomethacin-induced injury by increasing the expression of the tight junction proteins, and reducing JNK/Src phosphorylation [47]. Not surprisingly, it was reported that myricetin could alleviate DSS induced colitis via suppressing the TNF-α/NF-κB pathway, thereby increasing tight junction protein expression compared to colitis mice [54]. In addition, oral administration of myricetin-3-O-b-D-lactose sodium salt (M10), a derivative of myricetin, also exhibited preventive effect against ulcerative colitis through inhibiting the activation of IL-6 and TNF-α pathway, and phosphorylation of JAK2, STAT3, and NF-κB [55]. Herein, the results also indicated that M10 had higher efficacy than myricetin in the treatment of DSS-induced ulcerative colitis. Prior to that, Zhu et al. [127] also revealed similar results that M10 showed higher activities in preventing UC than myricetin.

#### 4.1.3. Flavanones

##### Hesperidin

Hesperidin (5,7,3′-trihydroxy-4′-methoxy-flavanone-7-rhamnoglucoside), belonging to the flavanone family, exists widely in citrus fruits and juices [128]. It was demonstrated that hesperidin had favorable effects on the intestine due to its antioxidant and anti-inflammatory activities [56,129,130]. For instance, hesperidin treatment ameliorates DSS-induced colitis and protects against intestinal inflammation through activating the nuclear factor E2-related factor 2 (Nrf2) antioxidant pathway and restoring intestinal barrier function [131]. A study conducted by Polat et al. [56] demonstrated that hesperetin administration significantly reduced colonic levels of NF-κB, TNF-α, and IL-6, thereby protecting the mice against TNBS-induced colitis. Alternatively, hesperidin methyl chalcone, the methylation process of hesperidin with higher water solubility, significantly reduced TNF-α, IL-6, IL-1β, and IL-33 production and inhibited NF-κB activation as observed by an increase in the total p65/phosphorylated-p65 ratio in a mouse model of acetic acid-induced colitis [57].

##### Naringenin

Naringin (4′,5,7-trihydroxyflavanone) extracted from citrus peels and grapefruit has been reported to exhibit various biological effects. Therein, some pieces of evidence show that naringin had beneficial effects on the intestinal barrier and amelioration of colitis [132,133,134]. In detail, naringin improved impaired intestinal permeability, inhibited the release of TNF-α and IL-6, and the expression of NF-κB, and thereby alleviated sepsis-induced intestinal mucosal injury [58]. Naringin supplementation reduced the development of colitis induced by DSS in mice through suppression of epithelial TNF-α production [133]. Moreover, a study performed by Ha et al. [59] also demonstrated that naringin inhibited the LPS-mediated activation of NF-κB and MAPKs pathways, and downstream COX-2, IL-1β, and TNF-α expression in macrophages.

#### 4.1.4. Flavanols

##### Epigallocatechin-3-Gallate (EGCG)

Tea, derived from the leaves of *Camellia sinensis*, is one of the most widely consumed beverages worldwide. EGCG, a predominant component of green tea polyphenols, is indicated to be primarily responsible for the anti-inflammatory and antioxidant effects of green tea [135]. Previously, a study conducted by Navarro-Perán et al. [136] demonstrated that EGCG could suppress TNF-α-induced NF-κB activation in colon cancer cells. In a high-fat diet-induced nonalcoholic steatohepatitis model in mice, EGCG significantly attenuated intestinal inflammation by decreasing ileal and colonic *TNF-α* expression and preventing the loss in expression of intestinal tight junction proteins [60]. EGCG inhibited LPS-induced IκBα degradation and NF-κB nuclear translocation in rat intestinal epithelial cells, thus suppressing adhesion molecules expression, indicating the therapeutic potential of EGCG on intestinal inflammatory diseases [61]. Moreover, EGCG prevented LPS-induced pro-inflammatory gene expression through blocking NF-κB and MAPK signaling pathways in bone marrow-derived macrophages [62].

#### 4.1.5. Isoflavones

##### Genistein

Genistein (4′,5,7-trihydroxyisoflavone) is a kind of natural phytoestrogens and isoflavones richly found in soybeans. Numerous in vitro and in vivo studies provided evidence that genistein plays an important role in the prevention and treatment of intestinal inflammation [63,137,138,139]. A study performed by Lv et al. [140] demonstrated that adding genistein into the diet of chicks can ameliorate LPS-induced intestinal injury via altering the RNA expression profile. More specifically, genistein inhibited I-κB kinase/NF-κB signaling, MAPK cascade, and JAK-STAT pathway, thereby improving the growth performance of chicks. Not surprisingly, genistein reduced DSS-induced inflammation response via suppressing the activation of TLR4/NF-κB signaling in Caco-2 cells [64]. In addition, in LPS-induced macrophages, gamma-irradiated genistein exerted an anti-inflammatory property associated with inhibition of TLR4-mediated NF-κB and MAPK pathways [65].

#### 4.1.6. Anthocyanins

##### Cyanidin-3-glucoside (C3G)

Anthocyanin-rich extracts have exhibited anti-inflammatory activity in mouse colitis models [141]. Cyanidin-3-glucoside (C3G) is a kind of natural anthocyanin originated from *Aronia melanocarpa* berries belonging to the Rosaceae family, Queen Garnet plums (*Prunus salicina* Lindl.), and purple carrots, which has been proven to provide anti-inflammatory potential in TNBS-induced colitis mice, LPS-stimulated Caco-2 cellular monolayer inflammation [141], and DSS-induced inflammatory bowel disease in rats [142]. Tan et al. [143] summarized the potential mechanism of C3G against intestinal injury, indicating its important role in the TLR4/NF-κB mediated pathway. More specifically, pretreatment with C3G dose-dependently prevented TNF-α-induced NF-κB pathway activation, thereby inhibiting IL-6 and COX-2 expression [66]. Moreover, in TNF-α induced Caco-2 and human umbilical endothelial cells (HUVECs) coculture model, C3G prevented the translocation of NF-κB into the nucleus and inhibited leukocyte adhesion in a dose-dependent manner, which suggested that anthocyanins may contribute to the treatment of chronic gut inflammatory diseases [67]. Not surprisingly, C3G inhibited NF-κB phosphorylation, reduced mRNA expression of pro-inflammatory cytokines including *IL-1β*, *IL-6*, *IL-8*, *COX-2*, and *TNF-α*, and protein levels of apoptosis related genes in DSS-induced colitis mice, providing new ideas for using C3G as adjuvant agent for treating UC [68].

##### Malvidin 3-glucoside (MV3G)

Malvidin 3-glucoside, one of the major anthocyanins present in blueberries, has been proven to possess antioxidant and anti-inflammatory function [69,144]. A study conducted by Liu et al. [145] demonstrated the favorable effects and mechanism of malvidin 3-glucoside (MV3G) in alleviating gut dysfunction using a murine colitis model induced by DSS, and the results showed that MV3G could attenuate intestinal inflammation through increasing IL-10 expression, and modulating gut microbiome and metabolome, indicating the beneficial effects of MV3G in promoting intestinal homeostasis and health. In a TNF-α-induced inflammatory model in HUVECs, MV3G suppressed IκBα degradation and blocked the nuclear translocation of NF-κB p65 [69].

Furthermore, MV3G downregulated the expression of iNOS and COX-2 in a TNBS-induced colitis rat model [70]. Moreover, in an in vitro epithelial-endothelial co-culture model, MV3G suppressed TNF-α stimulated expression of adhesion molecules, leukocyte adhesion, *NF-κB* mRNA expression, and secretion of IL-8 and IL-6, indicating the potential anti-inflammatory activity for the management of chronic intestinal diseases [71].

##### Pelargonidin and Pelargonidin-3-O-glucoside (P3G)

Pelargonidin-3-*O*-glucoside (P3G) is a major anthocyanin isolated from raspberries and strawberries, thought to be beneficial for human health [146,147]. Some pieces of evidence indicated that administration of pelargonidin attenuated TNBS-induced colitis in a dose-dependent manner [72]. To be specific, treating mice with TNBS increased the colonic expression of IL-6, TNF-α, IL-1β, and IFN-γ, colitis score, and intestinal permeability; this was fully reversed by pelargonidin administration [72]. In the study performed by [75], LPS stimulation for 1 h markedly promoted phosphorylation and degradation of IκBα, nuclear translocation of NF-κB p65, and phosphorylation of JNK, but this pattern was suppressed when macrophages were pretreated with P3G. Pretreatment with P3G also reduced 11 pro-inflammatory cytokines’ secretion, including IL-1α, TNF-α, IL-27, and IL-6, and enzymes (COX-2 and iNOS) related to inflammation in LPS-induced macrophages [75]. Similarly, P3G exhibited anti-inflammatory effects in LPS induced macrophages on account of arrest of the IκBα and NF-κB activation and reduction in JNK and p38 MAPK phosphorylation [76].

### 4.2. Phenolic Acids

#### 4.2.1. Caffeic Acid and Caffeic acid Phenethyl Ester (CAPE)

Caffeic acid is one of the most abundant hydroxycinnamic acids widely distributed in vegetables, fruits, and some beverages, such as potatoes, gooseberries, artichokes, and coffee [148]. It was indicated that caffeic acid can reach appropriate concentration in the colon where it could act on the intestinal cells and achieve its anti-inflammatory effects [74]. More than a decade ago, mice consuming caffeic-acid-enriched diets exhibited attenuation of DSS-induced colitis [149]. Correspondingly, caffeic acid exerted anti-inflammatory effects in DSS colitis mice associated with the inhibition of the NF-κB signaling pathway and suppression of the secretion of IL-6, TNF-α, and IFN-γ [73], which is similar with the results of [150]. In the study conducted by Zielińska et al. [74], IL-1β-stimulated myofibroblasts of the colon were employed as a human intestinal inflammation model. The results found that caffeic acid could reduce the expression of COX-2 and IL-8. In addition, CAPE, a biologically active ingredient of honeybee propolis, showed protective effects in treatment of DSS-induced colonic fibrosis [151] and intestinal ischemia-reperfusion injury [152]. In an ionized radiation-induced intestinal injury model in rats, pretreatment of CAPE reduced intestinal epithelial cell apoptosis, plasma TNF-α level, and phosphorylation of p38MAPK [77]. Recently, in a DSS-induced UC in a mouse model, administration of CAPE protected against colon damage by decreasing the expression of NF-κB and production of key cytokines [78].

#### 4.2.2. Chlorogenic Acid (CGA)

Chlorogenic acid (CGA) is a polyphenol compound present in various fruits, vegetables, and plants, such as honeysuckle, *Eucommia ulmoides*, coffee, and tea [153,154]. CGA has shown many biological effects including antioxidation, anti-inflammatory, anticancer, and antibacterial action [155,156]. Many in vitro and in vivo investigations reported that CGA can alleviate intestinal injury and inflammation [157,158,159]. For instance, CGA was shown to attenuate DSS-induced colitis in mice through the MAPK/ERK/JNK pathway [81]. Moreover, Vukelić et al. [160] also found that CGA can suppress the expression of ERK1/2, JNK1/2, STAT3, and nuclear translocation of NF-κB p65 for the purpose of ameliorating DSS-induced colitis. A study performed by Chen et al. [79] revealed that chlorogenic acid attenuated diquat-induced intestinal injury in weaned pigs associated with reduction in inflammatory cytokine secretion, and suppressed TNF-α-induced inflammation in IPEC-J2 cells via decreasing the phosphorylation of NF-κB and IκBα. CGA blocked the NF-κB pathway by preventing phospho-p65 translocation into cell nuclei, and suppressed TNF-α, IL-1β, and IL-6 production, and thereby restored intestinal epithelial tight-junction integrity [80]. It was also demonstrated that CGA could attenuate colonic barrier damage and promote dynamic distribution of tight junction proteins in TNBS-induced colitic rats [161]. CGA could be a promising medical countermeasure for the alleviation of intestinal inflammation.

#### 4.2.3. Ellagic Acid (EA)

Ellagic acid (EA), found in pomegranate (*Punica granatum* L.), has shown to exert anti-inflammatory and antioxidant properties. In this context, EA-enriched pomegranate extract markedly decreased COX-2 and iNOS overexpression, reduced MAPKs phosphorylation, and prevented nuclear NF-κB translocation, thereby attenuated chronic colonic inflammation [84]. In an ulcerative colitis model induced by acetic acid in rats, EA administration decreased the protein levels of TNF-α, COX-2, and NF-κB, and thereby exerted protective effects on colonic inflammation [82]. In the acute DSS-induced mice colitis model, EA attenuated colitis severity slightly through the reduction of inflammatory mediators (IL-6, TNF-α, and IFN-γ) [83]. Moreover, EA inhibited the NF-κB, p38 MAPK, and STAT3 signaling pathway, and enzymes related to inflammation, such as COX-2 and iNOS [83]. This pattern provides evidences that EA could be used in the dietary prevention of intestinal inflammation. Furthermore, urolithins, which are microbial metabolites of ellagic acid, have been widely reported in intestinal anti-inflammatory activity. In the DSS-induced rat colitis model, the author reported that urolithin-A decreased inflammation markers (iNOS and COX-2) and positively modulated the gut microbiota [162]. A study conducted by González-Sarrías et al. [163] revealed that urolithin-A is the main compound responsible for the EA anti-inflammatory properties, which is evidenced by its inhibitory effects on the activation of NF-κB and MAPK, and COX-2 expression in IL-1β-treated human colonic fibroblasts. Similarly, urolithin-A ameliorated cytokine-induced inflammation in human colon fibroblasts via downregulation of the levels of IL-8 and phenyl glycidyl ether E2 (PGE_2_), as well as cell migration and adhesion [164]. Some studies also revealed the protective effects of urolithin-A on gut barrier integrity [165,166]. Taken together, whether the intestinal inflammatory effects of EA are due to its microbiota-derived urolithins requires further characterization. 

### 4.3. Stilbenes

#### Resveratrol

Resveratrol (3,5,4-trihydroxy-trans-stilbene) is a polyphenolic compound found in peanuts, grape skins, and red wine [167]. Due to its multiple pharmacological activities, such as anti-inflammatory, antioxidant, and antitumor properties, it has been proven to be effective in a variety of inflammatory diseases, such as arthritis [168], pancreatitis [169], and UC [170,171]. Multiple lines of evidence indicate that resveratrol could alleviate intestinal injury and inflammation [85,86,87]. Additionally, an earlier study demonstrated that resveratrol could inhibit TLR4-mediated NF-κB activation through inhibiting TRAF6, and thus inhibiting JNK and p38 MAPK activation [172]. With our current knowledge, resveratrol could inhibit NF-κB activation and COX-2 expression in RAW264.7 cells following TLR4 stimulation [173]. Under circular heat stress, resveratrol reduced the protein expression of NF-κB and heat shock proteins (HSPs) in the jejunal villi, thereby alleviating jejunum mucosa injuries [174]. Resveratrol also reduced intestinal pro-inflammatory cytokine production including IL-1β, IL-6, and TNF-α, and downregulated the MAPK signaling pathway in post-weaning piglets [175]. More importantly, 6 weeks supplementation with 500 mg resveratrol can alleviate UC in patients associated with reduction in plasma levels of TNF-α and activity of NF-κB in peripheral blood mononuclear cells (PBMC) [176]. 

### 4.4. Other Polyphenols

#### 4.4.1. Curcumin

Curcumin, a natural active component extracted from the root of turmeric, a rhizomatous herbaceous perennial plant of the ginger family, is widely known to possess anti-inflammatory and antioxidant effects [88]. Previously, numerous studies in both animals and cell lines have demonstrated the inhibitory activity of curcumin on TLR4/MyD88/NF-κB signaling [89,90,177,178]. In intracolonic acetic acid-induced intestinal diarrhea and cold water induced constipation rat models, curcumin showed inhibitory effects on the NF-κB pathway by suppressing IκBα degradation and NF-κB phosphorylation [91]. IκBα inhibits NF-κB activation via forming an inactive NF-κB/IκBα complex [92]. It also attenuated experimental colitis induced by intra-rectal administration of TNBS through inhibition of TLR4 receptor, MyD88, and NF-κB protein expression [179]. 

#### 4.4.2. Emodin/Rhein

Emodin/rhein (1,3,8-trihydroxy-6-methyl-9,10-anthraquinone) is a natural anthraquinone compound that derives from many Polygonaceae plants, such as *Rheum officinale* Baill. There has been growing evidence showing that emodin with multiple pharmacological effects may be a promising agent for UC treatment [180,181,182]. Chen et al. [183] conducted a trial to investigate whether emodin can protect the jejunum against sepsis injury by inhibiting inflammation. As expected, the results found that emodin alleviated jejunum injury and inflammation via activating the JAK1/STAT3 signaling pathway, and decreasing the levels of IL-6 and TNF-α in septic rats. After that, it was observed that emodin markedly downregulated the expression of TLR5 and NF-κB p65 in the colon of DSS-induced colitis mice [182]. Besides, it also increased the expression of IκB, but inhibited the expression of TLR5 and MyD88, nuclear translocation of NF-κB p65, as well as the IL-8 production in flagellin-stimulated HT-29 cells [182]. In vitro, emodin led to inactivation of TLR4, NF-κB, and NLRP3, and also inhibition of IL-1β and IL-6 production, thereby exerting protective effects against barrier disruption and inflammation in an IEC-6 cell model with TNF-α stimulation, indicating potential therapeutic effects against intestinal diseases [181]. 

## 5. Conclusions and Future Perspectives

Polyphenols are a huge and various group of natural compounds of which only a few have been investigated regarding their alleviative effect on intestinal inflammation. This review summarized the intestinal anti-inflammatory properties of more than 20 kinds of polyphenols associated with modulation of the TLR4/NF-κB-mediated signaling pathway. It should be noted that the mechanisms for ameliorating intestinal inflammation are pleiotropic and usually target multiple sites of action in the TLR4/NF-κB signaling pathway, some of them are common between different polyphenols. In this regard, the listed polyphenols, collectively, inhibit the TLR4 receptor activation, and block the nuclear translocation of NF-κB, thereby reducing the production of downstream pro-inflammatory cytokines, such as IL-1β, IL-6, IL-8, TNF-α, and IFN-γ, and inflammation related enzymes, such as COX-2 and iNOS. Moreover, besides their inhibitory effect on TLR4/NF-κB cascade, these mentioned polyphenols also inhibit MAPK and JAK/STAT signaling pathways, which further confirmed their intestinal anti-inflammatory properties. This review provides evidence that polyphenols targeting the TLR4/NF-κB signaling pathway might be an effective approach or adjuvant agent to treat IBD in future clinical research implications. 

Alterations in chromatin play a vital role in pathological processes via regulating gene transcription [184]. Epigenetic processes with no changes to the DNA sequences mainly include DNA modifications, histone post-translational modifications (PTMs), microRNAs (miRNAs), and chromatin remodeling [185]. A recent review has summarized how polyphenols ameliorate various inflammatory diseases via epigenetic modification [186]. Although this review covered multiple polyphenols applied in various in vitro and in vivo inflammatory models for investigating their epigenetic regulatory mechanisms, few studies have focused on the epigenetic-mediated actions of these polyphenols to intestinal inflammatory models. Hence, in-depth investigations to reveal these polyphenols attenuating IBD associated with epigenetic alterations may help in finding new therapeutic targets for treating IBD. To the best of our knowledge, post-transcriptional modifications in RNA may have regulatory effects on different signal transductions [184]. In this respect, it will be of great benefit if further research is directed towards revealing how these polyphenols differentially regulate inflammatory-related miRNAs, and how they finally ameliorate the development of IBD. Furthermore, no studies report the effect of polyphenols on histone acylation. This lack of information highlighted the necessity of investigating the mechanisms by which polyphenols intervene in epigenetic modification. In addition to epigenetic regulations, most of the polyphenols containing a number of phenolic hydroxyl groups present low water solubility and are poorly absorbed in the small intestine, which may result in a great deal of differences in the results of in vivo and in vitro models. Therefore, the poor bioavailability of multiple polyphenols is another problem to be solved in further investigations. In this context, exploring nano-emulsion and nanoparticles formulations for polyphenols would be beneficial to improve the bioavailability of polyphenols [187]. More importantly, the anti-inflammatory effects of polyphenols must depend greatly on pharmacokinetics and cell access [38]. A substantial body of evidence has elucidated the pharmacokinetic profile of polyphenols. For example, quercetin glycosides are substrates of the intestinal glucose transporter (SGLT-1) in the rat, which may promote their absorption in the small intestine [188]. It was reported that flavanones, such as hesperidin and naringenin, can be taken up by epithelial cells through a H^+^-linked transporter and transcellular passive diffusion, thereby absorbed from the gastrointestinal tract [189,190,191]. Investigating pharmacokinetic variations between different polyphenols could help to further explore various combinations of polyphenols with similar absorption rates and distribution sites, and examine any potentiation of intestinal anti-inflammatory effects resulting from such combinations. On the other hand, it should be noted that polyphenols may exert anti-inflammatory effects in a dose-dependent manner. That is, increasing evidences indicate that polyphenols may show toxicity when used at higher concentrations [41,67,118]. Therefore, it is inevitable to explore effective technologies for enhancing bioavailability of several polyphenols at lower doses, such as solubilizers, targeted drug-delivery systems [192], and aforementioned nanotechnology. Furthermore, the anti-inflammatory effects of polyphenols are also dependent on the catabolites derived from the microbiota. From this perspective, the fermentation of phenolic compounds is an important issue that might be taken into consideration when investigating their beneficial effects. As stated in this review, numerous studies reported the intestinal anti-inflammatory effects of a single phytochemical substance; few studies investigated the interactions occurring between polyphenols [193]. Further work should therefore be conducted to investigate the polyphenol-polyphenol interactions and the combined effects of these interactions during intestinal inflammation. It has to be mentioned that pharmacokinetics of polyphenols should be taken into account when addressing the interactions due to the discrepancies in absorption, distribution, metabolism, and excretion inside the body [194,195].

## Figures and Tables

**Table 1:** Summary of polyphenols’ effects on intestinal inflammatory diseases along the TLR4/NF-κB-mediated signaling pathway in vitro and in vivo.

Polyphenol	Cell Type or Animal Model	Induction of Intestinal Inflammation	Anti-Inflammatory Mechanism	References
Apigenin	Swiss albino mice	Radiation-induced gastrointestinal damages	It inhibited NF-κB expression	Begum et al. [40]
	HCT-116 human colonic epithelial cancer cells	5 μg/mL LPS	It downregulated NF-κB and STAT3 expression, as well as IL-6 and IL-10 secretion in a dose dependent manner	Ai et al. [41]
	C57BL/6J mice	Oral administration of 1% DSS for 21 d	It reduced the severity of colitis by decreasing TNF-α, IL-1β, IL-6, and COX-2 levels	Ai et al. [41]
Luteolin	Human Caco-2 cells	5 μmol/L decabromodiphenyl ether (BDE-209) for 12 h	It inhibited ERK and NF-κB p50 expression and IκBα phosphorylation, as well as secretion of TNF-α, IL-6, IL-1β	Yuan et al. [42]
	C57BL/6J mice	Drinking water containing 3.0% DSS	It decreased the levels of IL-6, IL-1β, and TNF-α in the serum and colon, and the protein levels of TLR4, MyD88, and NF-κB p65, and phosphorylation of NF-κB p65	Zuo et al. [43]
	Caco-2/RAW264.7 co-culture model	LPS stimulation	It suppressed NF-κB nuclear translocation, and mRNA expression of *IL-8* and *TNF-α*	Nishitani et al. [44]
Baicalein	Female Balb/c mice	2 mg of TNBS	It reduced TNF-α and IL-1β, and phosphorylation of NF-κB p65 and IκBα, and protein expression of TLR4 and MyD88	Luo et al. [45]
	Sprague-Dawley rats	Ulcerative colitis	It inhibited NF-κB and MAPK expression, as well as IL-1β, IL-6, and IL-17	Liang et al. [46]
Quercetin	IEC-6 cells	300 μmol/L indomethacin for 24 h	It suppressed calcium-mediated JNK and Src activation	Fan et al. [47]
	Human intestinal epithelial cell line Int407	*Vibrio cholerae*	Pretreatment with it reduced the IL-8 secretion and NF-κB translocation into the nucleus	Das et al. [48]
	Male Sprague-Dawley rats	Acute necrotizing pancreatitis induced by 3.5% sodium taurocholate solution	It downregulated intestinal protein expression of TLR4 and MyD88, and phosphorylation of p38 MAPK	Zheng et al. [49]
	Sprague-Dawley rats	Indomethacin dissolved in 5% NaHCO_3_, at 40 mg/kg body weight	Its oxidation metabolite prevented NF-κB activation and IL-8 secretion	Fuentes et al. [50]
Kaempferol	Rat intestinal microvascular endothelial cells	10 µg/mL LPS for 12 h	It inhibited LPS-induced NF-κB, I-κB and STAT phosphorylation, decreased TLR4 overexpression, and LPS-induced IL-1β, IL-6 and TNF-α upregulation	Bian et al. [51]
	C57BL/6J male mice	High fat diet	It reduced the protein expression of TLR4, MyD88 and NF-κB, and mRNA expression of *TNF-α* in the colon	Bian et al. [52]
Rutin	Rag1 −/− mice	CD4^+^ CD62L^+^ T cells transfer model of colitis	It inhibited STAT4 and IκBαphosphorylation, as well as IL-1β and IFN-γ expression in CD4^+^ spleen cells of the mice	Mascaraque et al. [1]
	Female Wistar rats	10 mg of TNBS induced ileitis and colitis	Intragastric rutin resulted in reduced *IL-1β* and *IL-17* mRNA expression in the treatment of ileitis rats, while just tended to decrease levels of IL-17 and IFN-γ in the colitis rats	Mascaraque et al. [53]
Myricetin	IEC-6 cells	300 μmol/L indomethacin for 24 h	It increased the expression of tight junction proteins, and reduced JNK/Src phosphorylation	Fan et al. [47]
	Male Kunming mice	Oral administration of 3% DSS solution for 2 weeks	It suppressed TNF-α, NF-κB and COX-2 expression, and increased tight junction proteins expression	Li et al. [54]
Myricetin-3-O-b-D-lactose sodium salt	Male C57BL/6 mice	Oral water containing 1.0% DSS	It reduced the protein expression of IL-6, and the phosphorylation of JAK2, STAT3 and NF-κB, as well as TNF-α pathway, increased IL-4 and IL-10 secretion	Zhou et al. [55]
Hesperidin	Wistar albino male rats	TNBS-induced colitis	It reduced the colonic levels of NF-κB, TNF-α and IL-6	Polat et al. [56]
Hesperidin methyl chalcone	Male Swiss mice	Acetic acid-induced colitis	It reduced acetic acid-induced TNF-α, IL-6, IL-1β, and IL-33 production and inhibited NF-κB activation by blocking Ser276	Guazelli et al. [57]
Naringin	Mice	Cecal ligation and puncture-induced intestinal sepsis	It inhibited the release of TNF-α and IL-6, increased IL-10, inhibited NF-κB expression	Li et al. [58]
	RAW 264.7 macrophages	LPS (1 μg/mL /mL) stimulation for 24 h	It reduced NF-κB translocation and phosphorylation of p38, ERK, and JNK, as well as the expressions of COX-2, IL-1β and TNF-α	Ha et al. [59]
EGCG	Male C57BL/6J mice	High fat diet	It protected against gut barrier dysfunction, and decreased ileal and colonic mRNA expression of *TNF-α*	Dey et al. [60]
	Rat intestinal epithelial cells	LPS (1 μg/mL) stimulation for 24 h	It blocked NF-κB signaling via degradation of IκBα and inhibition of NF-κB nuclear translocation, thereby suppressed the expression of adhesion molecules ICAM-1 and VCAM-1	Myung et al. [61]
	Bone marrow-derived macrophages	LPS (1 μg/mL) incubation for 0–1 h	It prevented LPS-induced inflammation through inhibiting IκBα phosphorylation/degradation, NF-κB RelA nuclear translocation, and phosphorylation of ERK1/2, JNK and p38 expression	Joo et al. [62]
Genistein	Male Arbor Acre broilers	*Escherichia coli* O78	It improves intestinal mucosa barrier function by modulating apoptosis and secretion of TNF-α and IL-6	Zhang et al. [63]
	Caco-2 cells	3% DSS for 7 d	It reduced nuclear NF-κB p65 and upstream TLR4 expression	Zhang et al. [64]
	RAW 264.7 macrophage cells	LPS stimulation	It down-regulated TLR4 and NF-κB expression, IκBα degradation and phosphorylation of ERK1/2 and p38, as well as COX-2, TNF-α, IL-6 and IL-1β expression	Byun et al. [65]
Cyanidin-3-glucoside	Caco-2 cells	Exposed for 3 h to 50 ng/mL TNF-α	It inhibited NF-κB translocation into the nucleus, and IκBα degradation, as well as IL-6 and COX-2 expression	Ferrari et al. [66]
	Caco-2-HUVECs coculture model	Exposed for 1 h to 50 ng/mL TNF-α	It prevented translocation of NF-κB into the nucleus and inhibited leukocyte adhesion in a dose dependent manner	Ferrari et al. [67]
	Balbc mice	Drinking water containing 2.5% DSS	It suppressed NF-κB phosphorylation, thereby inhibited *IL-1β*, *IL-6*, *IL-8*, *COX-2* and *TNF-α* mRNA expression	Tan et al. [68]
Malvidin 3-glucoside	HUVECs	TNF-α (10 μg/L) stimulation for 6 h	It suppressed IκBα degradation and blocked the nuclear translocation of NF-κB p65	Huang et al. [69]
	Male Wistar rats	TNBS-induced colitis	It reduced leukocyte infiltration, downregulated iNOS and COX-2 expression	Pereira et al. [70]
	Caco-2-HUVECs coculture model	TNF-α (1 ng/mL) stimulation for 3h	It reduced *NF-κB* mRNA expression, and IL-8 and IL-6 secretion	Kuntz et al. [71]
Pelargonidin	Balb/c mice	TNBS-induced colitis	It decreased the colonic expression of IL-6, TNF-α, IL-1β, and IFN-γ, and increased IL-10 expression	Biagioli et al. [72]
	Female C57BL/6 mice	Drinking water containing 2.5% DSS for 8 d	It inhibited the activation of NF-κB p65 and IκBα degradation, as well as reduced the serum level of IL-6, IFN-γ and TNF-α	Zhang et al. [73]
	Myofibroblasts-like cell line	1 ng/mL IL-1β stimulation for 24 h	It reduced the IL-8 and COX-2 expression	Zielińska et al. [74]
Pelargonidin-3-*O*-glucoside	RAW 264.7 Macrophages	1 μg/mL LPS stimulation for 24 h	It inhibited nuclear translocation of NF-κB p65, phosphorylation and degradation of IκBα, as well as phosphorylation of JNK, thereby reduced the expression of pro-inflammatory cytokines, including IL-1α, TNF-α, IL-27, and IL-6, and enzymes related to inflammation, such as COX-2 and iNOS	Zhang et al. [75]
	RAW 264.7 Macrophages	1 μg/mL LPS stimulation for 24 h	It suppressed phosphorylation of JNK, p38 MAPK, IκBα and NF-κB p65, and reduced TNF-α and IL-6 production	Duarte et al. [76]
Caffeic acid phenethyl ester	Male Sprague-Dawley rats	X-ray irradiation (9 Gy)	It reduced the plasma level of TNF-α, and phosphorylation of p38MAPK	Jin et al. [77]
	Male Balb/c mice	Drinking water containing 3.5% DSS for 7 d	It reduced the production of key cytokines and expression of NF-κB p65	Pandurangan et al. [78]
Chlorogenic acid	IPEC-J2 cells	50 ng/mL TNF- α for 3 h	It inhibited the phosphorylation of NF-κB p65 and IκBα	Chen et al. [79]
	Caco-2 cells	LPS (0.1 mg/mL) stimulation for 24 h	It blocked nuclear translocation of NF-κB p65, and suppressed TNF-α, IL-1β and IL-6 production	Yu et al. [80]
Ellagic acid	C57BL/6 mice	Drinking water containing 5% DSS for 7 d	It reduced the protein expression and phosphorylation of ERK1/2, p38, and JNK	Gao et al. [81]
	Wistar Albino rats	3% acetic acid (2 mLintrarectal) induced colitis	It decreased the protein levels of TNF-α, COX-2, and NF-κB	Yipel et al. [82]
	Female Balb/C mice	Drinking water containing 5% DSS for 7 d	It reduced the production of IL-6, TNF-α, and IFN-γ	Marín et al. [83]
	Female C57BL/6 mice	Four week-long cycles of DSS (1% and 2%)	It inhibited p38 MAPK and STAT3 phosphorylation, IκBα degradation, NF-κB p65 activation, as well as IL-6, COX-2 and iNOS expression	Marín et al. [83]
	Four-week-old male Wistar rats	TNBS-induced colitis	It decreased the expression of TNF-α, COX-2, and iNOS, and p38 MAPK, p-JNK and p-ERK1/2, as well as the nuclear translocation of NF-κB p65	Rosillo et al. [84]
Resveratrol	Black-boned chickens	Circular heat stress	It reduced the jejunal protein expression of NF-κB	Liu et al. [85]
	Weaned piglets	Weaning stress	It downregulated MAPK pathway and reduced the levels of intestinal pro-inflammatory cytokines including IL-1β, IL-6, and TNF-α	Meng et al. [86]
	50 eligible patients	Ulcerative colitis	It reduced plasma levels of TNF-α and activity of NF-κB in peripheral blood mononuclear cells (PBMC)	Samsami-kor et al. [87]
Curcumin	Male Sprague-Dawley rats	Diarrhea and constipation induced by intracolonic acetic acid instillation or cold water gavage	It inhibited IκBα degradation and NF-κB phosphorylation, as well as IL-1β and TNF-α	Yao et al. [88]
	Male Sprague-Dawley rats	Experimental colitis induced by intra-rectal administration of TNBS	It Inhibited TLR4, MyD88 and NF-κB protein expression	Lubbad et al. [89]
Emodin	IEC-6 cells	TNF-α (50 ng/mL) stimulation	It inhibited the expression of TLR4, NF-κB and NLRP3, also the production of IL-1β and IL-6	Zhuang et al. [90]
	HT-29 cells	Flagellin (500 mg/L) stimulation for 24 h	It increased the expression of IκB, but inhibited the expression of TLR5 and MyD88, nuclear translocation of NF-κB p65, as well as the IL-8 production in flagellin-stimulated HT-29 cells	Luo et al. [91]
	Male Wistar rats	Cecal ligation and puncture induced jejunal sepsis	It decreased the levels of IL-6 and TNF-α, and increased the phosphorylated levels of JAK1 and STAT3	Chen et al. [92]
